# Vaginal Cuff Length and Time to Postoperative Stump Recurrence After Hysterectomy for Cervical Cancer: An Exploratory Single-Institution Study

**DOI:** 10.3390/jcm15197653

**Published:** 2026-10-02

**Authors:** Yuanjing Wang, Siqi Guan, Ming Wang, Yin Lv

**Affiliations:** 1 Department of Radiation Therapy Center, Beijing Obstetrics and Gynecology Hospital, Capital Medical University, Beijing Maternal and Child Health Care Hospital, Beijing 100006, China; 2 Department of Gynecological Oncology, Beijing Obstetrics and Gynecology Hospital, Capital Medical University, Beijing Maternal and Child Health Care Hospital, 17 Qihelou St., Dongcheng District, Beijing 100006, China

**Keywords:** cervical cancer, radical hysterectomy, stump recurrence, prognosis, vaginal cuff length, time to recurrence

## Abstract

**Backgroud/Objectives**: In this exploratory study, we explored the relationship between the timing of recurrence and vaginal cuff length (VCL) in patients with vaginal stump recurrence after hysterectomy. **Methods**: This is a single-institution study which included 25 consecutive patients who developed postoperative stump recurrence of cervical cancer at Beijing Obstetrics and Gynecology Hospital, Capital Medical University, between January 2013 and December 2024. Patients were divided into early recurrence (≤24 months) and late recurrence (>24 months) groups according to the postoperative 2-year cutoff point. The relationship between VCL and recurrence time was analyzed with Spearman rank correlation analysis. Receiver operating characteristic (ROC) curve analysis was performed to determine the optimal VCL cutoff value using the Youden index. Kaplan–Meier curves and log-rank tests were used to compare the time to recurrence between groups, stratified by the data-derived VCL cutoff. **Results**: The median age of the 25 patients was 57 years (range, 36–78 years). Squamous cell carcinoma accounted for 68.0% (17/25) and adenocarcinoma for 24.0% (6/25). The median VCL was 0.7 cm (range, 0.1–2.0 cm), and the median time to recurrence was 22 months (range, 5–89 months). Spearman correlation analysis showed a significant positive correlation between VCL and recurrence time (*r* = 0.804, *p* < 0.001). ROC curve analysis identified 0.75 cm as the optimal cutoff for predicting early recurrence, with an area under the curve (AUC) of 0.902, a sensitivity of 86.7%, and a specificity of 80.0%. Patients with VCL ≤ 0.75 cm (*n* = 15) had a median recurrence time of 15 months, compared with 43.5 months in those with VCL > 0.75 cm (*n* = 10). The proportions of patients remaining free of recurrence at 24 months were 13.3% and 80.0%, respectively (log-rank *p* < 0.001). **Conclusions**: In this study, shorter VCL was associated with earlier recurrence, and a data-derived cutoff of 0.75 cm discriminated early (≤24 months) from late recurrence. Further multicenter studies are needed to validate this finding before changing clinical decisions, given the restricted sample size.

## 1. Introduction

Cervical cancer is the fourth most common malignancy among women worldwide, with an estimated 604,000 new cases and 342,000 deaths reported in 2020, and its burden remains disproportionately high in low- and middle-income countries [1,2]. China alone contributes approximately one fifth of the global cervical cancer burden [3], and marked geographic and socioeconomic disparities in incidence and outcomes persist worldwide [4]. For patients with International Federation of Gynecology and Obstetrics (FIGO) 2018 stage IB–IIA disease, radical hysterectomy with pelvic lymphadenectomy remains the standard surgical treatment, achieving 5-year overall survival rates of approximately 90% in early-stage disease [5,6,7]. Nevertheless, 10–20% of patients experience recurrence after primary surgery, and recurrence remains the principal cause of treatment failure and cancer-related death in this population [8].

Approximately 70–80% of postoperative recurrences develop within the first 2 years after surgery [9,10]. Among the patterns of failure, the vaginal stump (vault) is a common site of locoregional recurrence after hysterectomy. Once vaginal recurrence occurs, the salvage radiotherapy or pelvic exenteration in highly selected cases offers the best chance of durable disease control when the recurrence is detected early and remains localized [10,11]. In extensive recurrent or metastatic disease, systemic treatment has been shown to improve survival, underscoring the value of detecting recurrence at a treatable stage [12]. Therefore, identifying factors associated not only with whether recurrence occurs but also with when it occurs carries direct clinical relevance for surveillance planning and timely intervention.

Radical hysterectomy is designed to achieve adequate resection of the parametrial tissue and the upper vagina to ensure oncological safety. However, consensus regarding the optimal length of vaginal resection has long been lacking, with traditional recommendations based largely on anatomical considerations rather than high-level evidence [13], and with neither the Piver classification [14] nor the Querleu–Morrow classification [15,16] prescribing an absolute resection length. A prospective randomized trial found no survival benefit from more extensive resection [17], yet excessive resection may compromise postoperative quality of life [18,19,20]. Despite its surgical relevance, VCL has not been incorporated into mainstream risk-stratification systems, which rely primarily on pathological factors such as nodal status, parametrial invasion, margin status, lymphovascular space invasion(LVSI), tumor size, and depth of stromal invasion [21,22,23,24,25]. For patients with high-risk factors, adjuvant concurrent chemoradiotherapy is recommended [26], which may obscure the prognostic weight of surgical parameters. Evidence regarding VCL itself remains conflicting [13,27], and adjuvant radiotherapy may partly compensate for inadequate margins [28,29]. Notably, previous studies have focused almost exclusively on the relationship between VCL and recurrence risk, whereas the association between VCL and the timing of recurrence remains unclear.

Notably, previous studies have focused almost exclusively on the relationship between VCL and the risk of recurrence, whereas the association between VCL and the timing of recurrence among patients who ultimately develop recurrence remains unclear. Given that most recurrences cluster within the first 2 postoperative years and that early recurrence generally indicates more aggressive tumor biology with a narrower window for salvage therapy, a parameter capable of distinguishing early from late recurrence could help refine individualized postoperative surveillance strategies. The aim of this exploratory, single-institution study was as follows: (i) to evaluate the association between VCL and the time to stump recurrence among recurrent cases; (ii) to identify an optimal VCL cutoff value for predicting early (≤2-year) recurrence; and (iii) to explore the potential—pending external validation—implications of VCL-based stratification for postoperative surveillance.

## 2. Materials and Methods

### 2.1. Study Design and Patients

This retrospective cohort study was conducted at Beijing Obstetrics and Gynecology Hospital, Capital Medical University, and is reported in accordance with the STROBE (Strengthening the Reporting of Observational Studies in Epidemiology) recommendations. Patients who developed postoperative stump recurrence after hysterectomy (including radical hysterectomy with pelvic lymphadenectomy or extrafascial hysterectomy) for cervical cancer between January 2013 and December 2024 were retrospectively enrolled. All patients were restaged according to the FIGO 2018 staging system, with stages ranging from IA1 to IIIC2p.

The inclusion criteria were: (1) pathologically confirmed cervical squamous cell carcinoma, adenocarcinoma, adenosquamous carcinoma, or clear cell carcinoma; (2) FIGO 2018 stage IA1–IIIC2p disease; (3) age ≥ 18 years; (4) having undergone hysterectomy (radical hysterectomy with pelvic lymphadenectomy or extrafascial hysterectomy); (5) postoperative stump recurrence confirmed by biopsy; and (6) complete clinical and follow-up over the 12-year accrual period with available VCL measurements. The exclusion criteria were: (1) neoadjuvant chemotherapy or radiotherapy before surgery; (2) concurrent other pelvic malignancies; and (3) positive or suspicious vaginal margins on final pathology; and (4) severe comorbidities, including uncontrolled diabetes mellitus or hereditary cancer syndromes, which could confound the analysis of recurrence timing.

This study was approved by the Institutional Review Board of Beijing Obstetrics and Gynecology Hospital, Capital Medical University (Approval No.: 2026-KY-072-02) on 5 June 2026, and written informed consent was waived for this retrospective study.

### 2.2. Surgical Procedures and VCL Measurement

Radical hysterectomy corresponded to Querleu–Morrow type B–C resection [15,16] with systematic pelvic lymphadenectomy, with or without para-aortic lymph node sampling, whereas extrafascial (type A) hysterectomy was performed in selected patients with early-stage disease. The choice of surgical approach was made by the attending gynecologic oncologist according to preoperative staging and intraoperative findings.

VCL was defined as the length of vaginal tissue resected distal to the tumor, measured from the distal tumor edge to the vaginal resection margin. All VCL measurements were performed by dedicated gynecologic pathologists on the resected specimens using a standardized protocol, and the vaginal resection margins were evaluated microscopically. All cases included in this study had pathologically negative vaginal margins.

### 2.3. Definitions and Follow-Up

Vaginal stump recurrence was defined as a recurrence tumor located at the vaginal stump (vault) after hysterectomy. Time to recurrence was defined as the interval in months from the date of surgery to the date of pathological confirmation of recurrence. Based on the well-established temporal distribution of postoperative recurrence in cervical cancer, recurrence occurring within 24 months after surgery was defined as early recurrence, whereas recurrence beyond 24 months was defined as late recurrence.

Postoperative surveillance was performed according to institutional protocols, consistent with contemporary guidelines, consisting of gynecologic examination and cytology every 3–4 months during the first 2 years, every 6 months during years 3–5, and annually thereafter. Pelvic magnetic resonance imaging, computed tomography, or positron emission tomography was performed when clinically indicated, and histological confirmation by biopsy was required for the diagnosis of recurrence in all cases.

### 2.4. Data Collection and Analysis

The following data were retrieved from electronic medical records and pathology reports: age, surgical approach (radical hysterectomy with pelvic lymphadenectomy vs. extrafascial hysterectomy), histological type (squamous cell carcinoma, adenocarcinoma, adenosquamous carcinoma, or clear cell carcinoma), maximum tumor diameter (cm), depth of stromal invasion (inner/middle/outer one-third), lymphovascular space invasion (positive/negative), VCL (cm), and time from surgery to pathologically confirmed recurrence (months). Patients were divided into short-resection and long-resection groups according to the optimal VCL cutoff determined by ROC curve analysis using the Youden index. The specific cutoff value and the sample sizes of each group are reported in Section 3.

### 2.5. Statistical Analysis

Statistical analyses were performed using SPSS 26.0 (IBM Corp., Armonk, NY, USA). Continuous variables (age, VCL, tumor size, and recurrence time) were assessed for normality with the Shapiro–Wilk test. Normally distributed variables were expressed as mean ± standard deviation and compared with the *t*-test; skewed variables were expressed as median (range) and compared with the Mann–Whitney U test. Categorical variables were expressed as frequencies (percentages) and compared using the χ^2^ test or Fisher’s exact test (expected frequencies < 5). Spearman rank correlation was used to assess the association between VCL and recurrence time, given the skewed distribution of recurrence time.

For ROC analysis, patients were stratified into early (≤24 months) and late (>24 months) recurrence groups; with this binary variable as the state variable and VCL as the test variable, the area under the curve (AUC), sensitivity and specificity were calculated, and the optimal VCL cutoff was determined by the maximum Youden index (sensitivity + specificity-1). Kaplan–Meier curves were constructed to describe the time to recurrence in the two VCL groups, with recurrence within 24 months as the event (event = 1) and administrative censoring at 24 months for patients who were recurrence-free by then (censored = 0). Because this study only included patients with tumor recurrence, the curves describe the distribution of time to recurrence in a recurrent cohort rather than a recurrence-free survival endpoint. Groups were compared using the log-rank test. A two-sided *p* < 0.05 was considered statistically significant.

## 3. Results

### 3.1. Baseline Characteristics

A total of 25 patients with postoperative stump recurrence were included in this analysis, and their clinicopathological characteristics are summarized in Table 1. The median age was 57 years (range, 36–78 years). The majority of patients had early-stage disease at initial diagnosis, with FIGO 2018 stage IA1 accounting for 32.0% (8/25) and stage IB1 for 28.0% (7/25). Squamous cell carcinoma was the most common histological type (68.0%, 17/25), followed by adenocarcinoma (24.0%, 6/25), adenosquamous carcinoma (4.0%, 1/25), and clear cell carcinoma (4.0%, 1/25). Surgical approaches included radical hysterectomy with pelvic lymphadenectomy (68.0%, 17/25) and extrafascial hysterectomy (32.0%, 8/25). The median tumor diameter was 1.8 cm (range, 0.2–5.0 cm), and the depth of stromal invasion was limited to the inner one-third in 48.0% (12/25) of cases. The median VCL was 0.7 cm (range, 0.1–2.0 cm), and the median time to recurrence was 22 months (range, 5–89 months). Overall, 15 patients (60.0%) developed recurrence within 24 months after surgery, whereas 10 patients (40.0%) experienced late recurrence. The small size of this single-institution cohort reflects the low incidence of vault recurrence and should be borne in mind when interpreting all subsequent analyses.

### 3.2. Correlation Between VCL and Time to Recurrence

Spearman rank correlation analysis revealed a significant positive correlation between VCL and time to recurrence (r = 0.804, *p* < 0.001), indicating that, among patients who had already developed recurrence, a shorter VCL was associated with earlier recurrence. The correlation coefficient of 0.804 suggests a strong monotonic relationship between the extent of vaginal resection and the chronology of recurrence in this cohort.

### 3.3. Roc Curve and Optimal Cutoff Value

**Figure 1:**
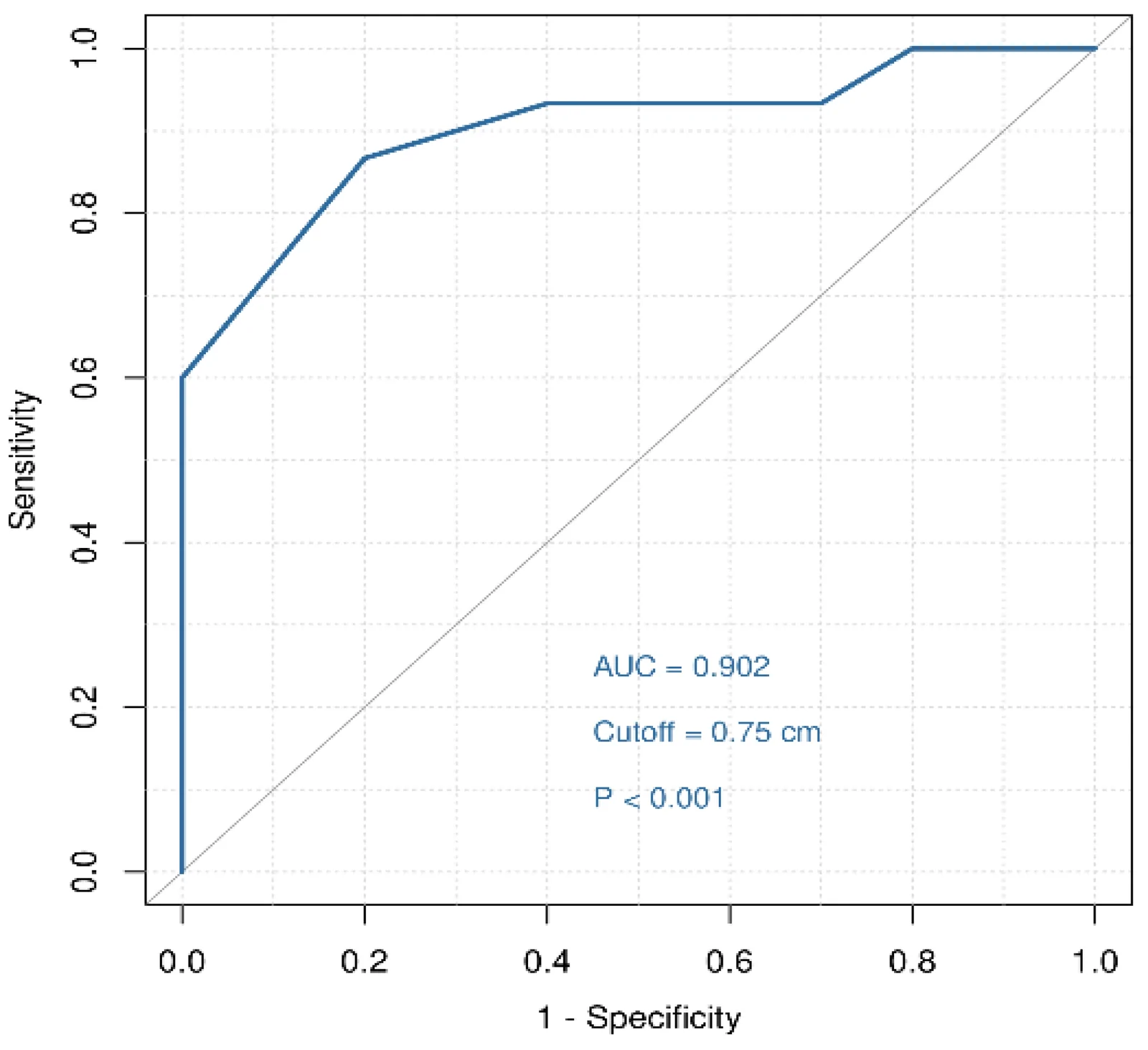
ROC curve of VCL for discriminating early (≤24 months) from late recurrence among patients who developed stump recurrence (AUC = 0.902; data-derived optimal cutoff = 0.75 cm).

ROC curve analysis was performed to evaluate the predictive value of VCL for recurrence within 2 years, with patients stratified by recurrence time (≤24 months vs. >24 months). The Youden index identified 0.75 cm as the optimal data-derived VCL cutoff, with an AUC of 0.902, a sensitivity of 86.7%, and a specificity of 80.0% (Figure 1). Because the cutoff was derived and evaluated in the same 25-patient dataset, this estimate of discriminatory performance is likely optimistic and requires external validation before any clinical use.

### 3.4. Comparison of Recurrence Time Between VCL Groups

**Figure 2:**
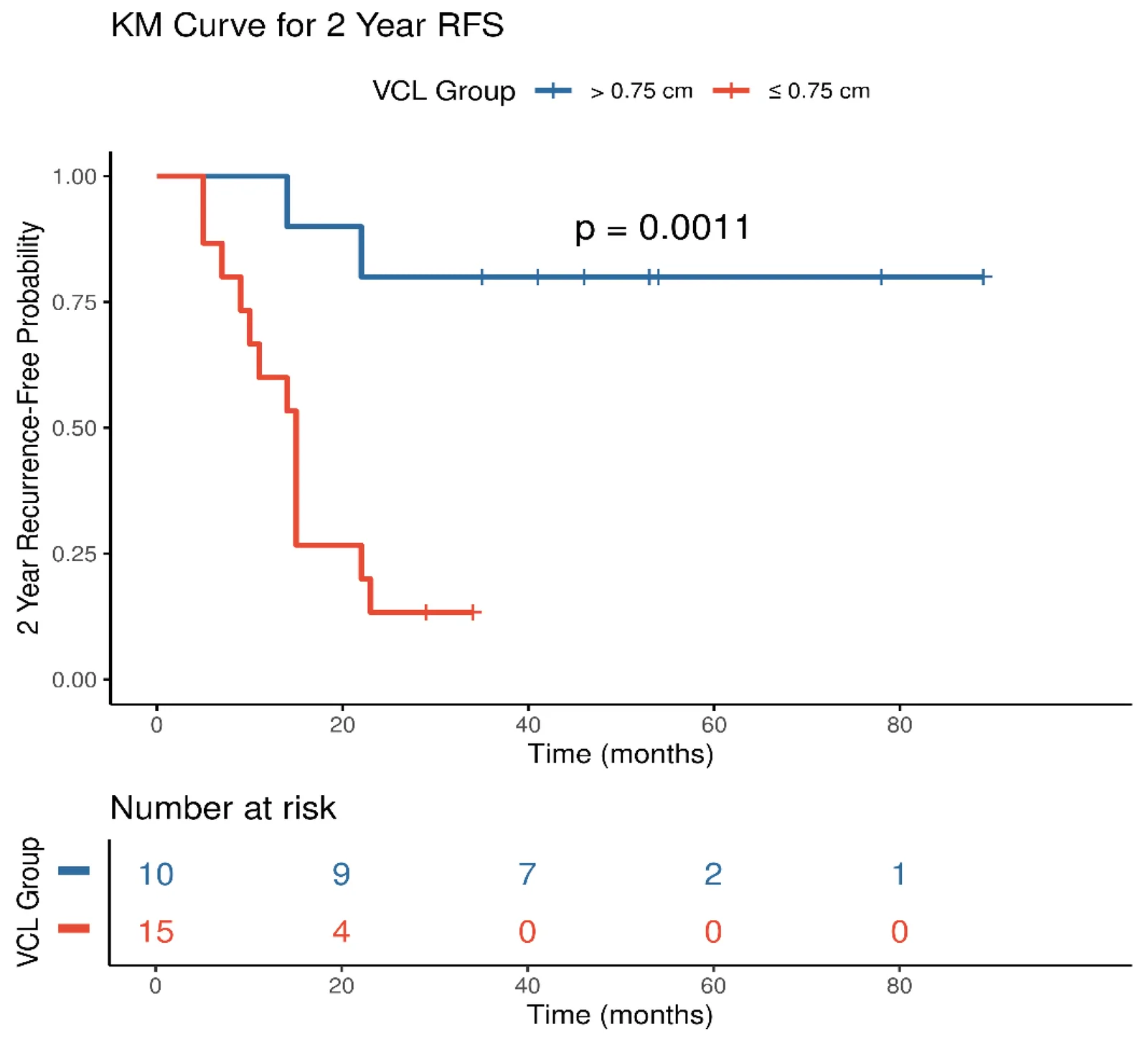
Kaplan–Meier curves of time to stump recurrence by VCL group (patients censored at 24 months). All included patients ultimately developed recurrence; the curves, therefore, depict the proportion still free of recurrence at each time point.

Using 0.75 cm as the cutoff, patients were divided into a short-resection group (VCL ≤ 0.75 cm, *n* = 15) and a long-resection group (VCL > 0.75 cm, *n* = 10). The short-resection group had a median recurrence time of 15 months, whereas the long-resection group had a median recurrence time of 43.5 months, corresponding to an almost threefold difference. The Kaplan–Meier analysis showed a significant difference in the time to recurrence between the two groups; the proportions of patients remaining free of recurrence at 24 months were 13.3% and 80.0%, respectively (log-rank *p* < 0.001) (Figure 2). To assess robustness to disease stage, a sensitivity analysis excluding the 8 FIGO IA1 patients (*n* = 17) preserved the VCL–recurrence-timing association: the optimal cutoff remained 0.75 cm, with a significant correlation and a persistent difference in the time to recurrence between VCL groups (Supplementary Table S2).

### 3.5. Univariate Analysis of Factors Associated with 2-Year Recurrence

The univariate analysis revealed no significant associations between age, histological type, or lymphovascular space invasion and recurrence within 2 years (all *p* > 0.05) (Table 2). A deeper depth of stromal invasion showed a trend toward a higher proportion of early recurrence, although this did not reach statistical significance (outer 1/3 vs. inner 1/3: 78.6% vs. 28.6%, *p* = 0.075). In contrast, VCL was significantly associated with early recurrence: patients with a VCL ≤ 0.75 cm had a markedly higher proportion of recurrence within 2 years compared with those with a VCL > 0.75 cm [86.7% (13/15) vs. 20.0% (2/10), *p* = 0.002], and VCL was the only variable significantly associated with 2-year recurrence in this cohort.

## 4. Discussion

### 4.1. Main Findings

Postoperative stump recurrence is a critical event affecting long-term survival in patients with cervical cancer, and the timing of recurrence carries important clinical implications. Early recurrence (within 2 years after surgery) generally suggests aggressive tumor biology or the presence of occult residual disease, leaving a narrower window for salvage therapy and being associated with a worse prognosis [8,11]. However, the relationship between VCL and the timing of stump recurrence has rarely been investigated. In this exploratory single-institution analysis with 25 patients, we found that a shorter VCL was associated with earlier recurrence, and 0.75 cm was identified as the optimal cutoff for distinguishing early (≤24 months) from late (>24 months) recurrence, with an AUC of 0.902, a sensitivity of 86.7%, and a specificity of 80.0%. However, these results are restricted to the sample size, and further studies are needed for the multivariable adjustment analysis and external validation before clinical practice.

### 4.2. Comparison with Previous Studies

In previous studies, Kim et al. [13] found no correlation between VCL and 3-year vaginal or pelvic recurrence rates in 280 patients undergoing radical hysterectomy. However, the endpoint of this study was the 3-year recurrence rate, and 68% of their patients received adjuvant therapy, which may have masked the independent effect of VCL. Zuo et al. investigated the relationship between VCL and recurrence risk in a large cohort including both recurrent and non-recurrent patients, in which the 2.0 cm threshold reflects the impact of surgical extent on whether recurrence occurs [27]. In this study, we found that the 0.75 cm cutoff further distinguished patients with early recurrence from those with late recurrence. Adjuvant radiotherapy with or without brachytherapy has been shown to compensate for inadequate or positive vaginal resection margins [28], and vaginal margin status has been more consistently established as a risk factor for recurrence than VCL itself [29]. These data collectively highlight the importance of timely adjuvant radiation in postoperative patients with short vaginal cuff length.

### 4.3. Potential Mechanisms

Several mechanism may explain the association between shorter VCL and earlier stump recurrence. First, vaginal mucosa adjacent to the cervix may harbor subclinical tumor extension or intraoperatively implanted cells. A shorter resection leaves limited clearance around such occult disease and permits earlier progression. A longer resection removes more involved mucosa, confining recurrence to later de novo events. Second, intraoperative implantation at the vaginal vault, promoted by tumor disruption and manipulator use, may seed the stump [30,31]. Third, paravaginal lymphatic microinvolvement may mediate early locoregional relapse independent of resection length. Fourth, early recurrence marks more aggressive tumor biology [8,11], and a shorter VCL retains more distal mucosa that may harbor HPV-driven field changes or occult lesions, providing a substrate for earlier manifestation. Testing these hypotheses will require pathological and molecular correlates, such as HPV genotyping and the profiling of tissue adjacent to the resection margin.

### 4.4. Clinical Implications

These findings provide a quantifiable reference for postoperative surveillance stratification. Current guidelines recommend intensive follow-up during the first 2 postoperative years without differentiating intensity by surgical parameters [9,10,23]. If validated externally in larger multicenter cohorts with adequate multivariable adjustment, VCL could support risk-adapted surveillance. Patients with a margin below 0.75 cm might benefit from timely adjuvant radiotherapy to reduce early recurrence, and those with longer margins might benefit from intensified surveillance, with vaginal cytology and HPV testing. In the present study, the cutoff was derived and evaluated within the same small dataset, without adjustment for adjuvant therapy, surgical approach, or pathological confounders, and these hypothesis-generating findings do not justify changes to guideline-based follow-up pending validation. This timing-based stratification complements risk factor-based adjuvant therapy decisions relying on nodal status, parametrial invasion, margin status, LVSI, tumor size, and stromal invasion depth [23,25] and aligns with recent studies supporting risk-adapted surveillance in recurrent cervical cancer [32]. Surgically, these findings support obtaining and documenting an adequate vaginal margin. Routine reporting of VCL in pathology reports would facilitate future research and individualized postoperative management [33].

### 4.5. Strengths and Limitations

The strengths of this study include the pathological confirmation of stump recurrence in all cases, standardized VCL measurement by dedicated gynecologic pathologists, uniformly negative vaginal margins—allowing assessment of the effect of VCL independent of margin status—and a long accrual period spanning more than a decade. To our knowledge, this is the first study to specifically examine the association between VCL and the timing of stump recurrence.

Several limitations should be acknowledged. First, the small sample size limited statistical power for multivariate analysis, and the 0.75 cm cutoff requires validation in larger cohorts. After excluding stage IA1 patients, sensitivity analysis supported the robustness of the association (Supplementary Table S2). Second, the cutoff was derived from and evaluated within the same dataset, so the reported diagnostic performance is likely optimistic, and external validation is essential before clinical application. Third, the inclusion of only patients with recurrence, without a non-recurrent control group, precludes assessment of the impact of VCL on overall recurrence risk. Fourth, the retrospective, single-center design may have introduced selection and information bias, partially offset by standardized pathological measurement procedures. Fifth, confounders including adjuvant therapy, surgical approach and histological diversity could not be adequately adjusted for. Finally, VCL measured on resected specimens may vary with tissue handling and fixation.

### 4.6. Future Directions

Large-scale, multicenter prospective cohort studies including both recurrent and non-recurrent patients are warranted to externally validate the 0.75 cm cutoff and to clarify the independent contribution of VCL to recurrence risk and timing after adjustment for adjuvant therapy and other pathological factors. Combining VCL with molecular and virological markers, such as HPV genotype and circulating tumor DNA, may further refine recurrence prediction, in line with emerging evidence on integrating surgical parameters with molecular biomarkers in cervical cancer survivors. A VCL-guided, risk-adapted surveillance strategy should then be evaluated prospectively to determine whether timely intervention and intensified follow-up in patients with short resections translate into earlier detection and improved survival.

## 5. Conclusions

In this single-institution study with a small sample size, shorter vaginal cuff length was associated with earlier recurrence; a data-derived 0.75 cm cutoff discriminated early from late recurrence. However, further multicenter studies with external validation and multivariable analysis are needed before these findings are used in clinical decision-making, given the restricted sample size.

## Figures and Tables

**Table 1:** Clinicopathological characteristics of patients with postoperative stump recurrence.

Characteristic	Value (N = 25)
Median age, years (range)	57 (36–78)
FIGO stage (2018 version)	
IA1	32.0% (8/25)
IB1	28.0% (7/25)
IB2	4.0% (1/25)
IB3	4.0% (1/25)
IIA1	12.0% (3/25)
IIIC1p	16.0% (4/25)
IIIC2p	4.0% (1/25)
Histological type	
Squamous cell carcinoma	68.0% (17/25)
Adenocarcinoma	24.0% (6/25)
Adenosquamous carcinoma	4.0% (1/25)
Clear cell carcinoma	4.0% (1/25)
Median tumor diameter, cm (range)	1.8 (0.2–5.0)
Depth of stromal invasion	
Inner 1/3	48.0% (12/25)
Middle 1/3	16.0% (4/25)
Outer 1/3	36.0% (9/25)
Surgical approach	
Extrafascial hysterectomy	32.0% (8/25)
Radical hysterectomy	68.0% (17/25)
Median VCL, cm (range)	0.7 (0.1–2.0)
Median time to recurrence, months (range)	22 (5–89)

**Table 2:** Univariate analysis of clinical factors associated with recurrence within 2 years among 25 patients with stump recurrence (no multivariable analysis was performed).

Clinical Characteristics	N	Recurrence Time		χ^2^	*p*
		≤2 Years	>2 Years		
Age (years)				-	1.000 a
≤60	14	8 (57.1%)	6 (42.9%)		
>60	11	7 (63.6%)	4 (36.4%)		
Pathological type				-	1.000 a
Squamous cell carcinoma	17	10 (58.8%)	7 (41.2%)		
Non-squamous cell carcinoma	8	5 (62.5%)	3 (37.5%)		
Lymphovascular space invasion					1.000 a
Positive	8	5 (62.5%)	3 (37.5%)		
Negative	17	10 (58.8%)	7 (41.2%)		
Stromal invasion					
Deep 1/3	14	11 (78.6%)	3 (21.4%)	-	0.075 b
Middle 1/3	4	2 (50.0%)	2 (50.0%)		
Superficial 1/3	7	2 (28.6%)	5 (71.4%)		
vaginal cuff length				-	0.002 a,*
≤0.75 cm	15	13 (86.7%)	2 (13.3%)		
>0.75 cm	10	2 (20.0%)	8 (80.0%)		

Note: a represents the *p*-value of Fisher’s exact tests; b represents the *p*-value of likelihood ratio tests; * denotes *p* values less than 0.05.

## Data Availability

The primary data supporting the findings of this study are available from the corresponding author on reasonable request. The data supporting the findings of this study are available in the Supplementary Material of this article (Supplementary Table S1).

## Supplementary Materials

The following supporting information can be downloaded at: https://www.mdpi.com/article/10.3390/jcm15197653/s1. Table S1. Clinicopathological characteristics of cervical cancer patients with postoperative vaginal stump recurrence (*n* = 25) VCL, vaginal cuff length; LVSI, lymphovascular space invasion. Time to recurrence (months) is the interval from the date of surgery to the date of histopathological confirmation of vaginal stump recurrence. Recurrence group: early = recurrence at ≤24 months after surgery; late = recurrence at >24 months. Table S2. Sensitivity analysis of the association between vaginal cuff length and recurrence timing after excluding the 8 patients with FIGO 2018 stage IA1 disease. AUC, area under the curve; FIGO, International Federation of Gynecology and Obstetrics; ROC, receiver operating characteristic; VCL, vaginal cuff length.
